# Using Strength and Risk Factors to Inform Treatment Typologies Over Time for Men on Community Supervision

**DOI:** 10.1177/0306624X211027485

**Published:** 2021-06-28

**Authors:** Kayla A. Wanamaker, Shelley L. Brown

**Affiliations:** 1 Carleton University, Ottawa, ON, Canada

**Keywords:** typologies, reoffending, strengths, latent transition analysis, community supervision

## Abstract

This study examines treatment typologies over time and their relationship to reoffending outcomes. Latent transition analysis was conducted with 6,675 men on community supervision in Alberta, Canada using risk and strength factors measured by the Service Planning Instrument (Orbis Partners, 2003). Three timepoints were assessed: Time 1 = first assessment within 90 days of start of supervision, Time 2 = 3 to 8 months post initial assessment, and Time 3 = 9 to 14 months post initial assessment. Five profiles consistently emerged: Low risk/Low strength profile, Aggressive, complex need/Low strength profile, Moderate risk/Moderate strength profile, Low risk/High strength profile, and Non-aggressive, complex need/Low strength profile. At Time 3, a sixth profile emerged labeled Moderate complex need/Low strength. Profiles characterized as aggressive and those with complex needs had highest rates of reoffending. Results demonstrate the utility of incorporating strengths, mental health needs, and adverse childhood experiences in risk assessment protocols.

Typology research has been prominent in correctional research, whereby individuals are grouped based on commonly shared characteristics. Often these typologies are based on the clustering of various risk factors. According to Jones and Harris (1999), there are four reasons to classify individuals into correctional typologies: (1) to help build our understanding of why people engage in criminal activity, (2) to aid in our approach to treatment and intervention protocols to improve responsivity, (3) to help guide case management, and (4) to help determine who are more likely to reoffend. To develop these typologies, studies have used numerous statistical techniques (e.g., multidimensional scaling, path analysis, latent class analysis (LCA), cluster analysis), which has increased the variability in the number of identified subtypes. To date, virtually no typology or strengths-based studies have examined how strengths may aid in the typological development of adults involved in the criminal justice system. Further, most typology studies have been cross-sectional in design and do not consider change over time. The current study will combine risks and strengths to examine typologies that emerge for men on community supervision and whether these typologies remain stable over time.

## Trajectory-Based Typologies

Trajectory-based typologies examine the development of criminal behavior over time (e.g., how it increases, decreases, stays the same) and focuses predominately on adolescence. One of the only trajectory-based studies to include strengths in the trajectory development was Baglivio et al. (2017). Using a sample of 6,442 youth in residential facilities assessed with the Residential Positive Achievement Change Tool (R-PACT; used by the Florida Department of Juvenile Justice), trajectories were developed based on youth’s buffer score progression. The buffer score assesses risk reduction while also considering strength enhancement (buffer = strength − risk). Notably, the R-PACT includes several risk and strength domains, including relationships with family and friends, substance use, mental health, attitudes, skills, ability to control impulses and aggression, etc.). Based on semi-parametric group-based modeling with four assessments (*n* *=* 4,870) and five assessments (*n* = 1,846), results indicated that youth in residential facilities progress through different buffering trajectories. Specifically, between six and seven trajectories emerged (depending on number of assessments). Groups were characterized by initial buffer score and buffer score changes during placement (e.g., Low initial—minimal gains; High initial—moderate gains; etc.). Trajectories which had the most improvement in buffer scores over time had the lowest recidivism rates, which illustrates the utility of incorporating strengths in typological research and risk assessment protocols, especially when considering changes over time. This study, however, did not characterize group membership based on similarities or differences in specific risk/needs and strengths.

## Treatment Typologies

In contrast, treatment typology studies, prevalent among correctional research, group individuals based on their risks or needs which can help inform effective correctional treatment interventions and aid in our understanding of how various risk/need factors operate together. These studies have found between four and five typologies of justice-involved individuals. Each of these studies have found a general low-risk subgroup and a general high-risk subgroup, as well as two or three subgroups defined by specific dynamic risk factors, such as personality or substance use deficits. These studies typically only include criminogenic need factors and do not include strengths. Gender-responsive treatment typology studies have also highlighted the importance of mental health and victimization among women and men (e.g., Jones et al., 2014).

One typology study that included an abundance of risk/need factors (e.g., substance use, educational issues, aggression, family issues, physical and sexual abuse, promiscuity, and socioeconomic status) to perform a multiple cluster analysis was Brennan et al. (2008). Using a sample of 1,572 justice-involved youth (72% male) from three jurisdictions in America, several clusters emerged. The Internalizing Youth A (*n* = 83) group is described as withdrawn, abused, and rejected delinquents. The Socially Deprived (*n* = 103) group is described as socially deprived delinquents from lower socioeconomic statuses. The Low Control A (*n* = 85) group is described as versatile, impulsive, low empathy, manipulative, and have negative school experiences and pro-criminal peers. The Normal “Accidental/Situational” Delinquents (*n* = 151) group described as youth who display limited risk factors, lower number of adjudications, and lower age at first adjudication. The Internalizing Youth B (*n* = 197) group was similar to Internalizing Youth A, but had significantly higher number of violent charges and did not come from abusing or neglectful households. The Low Control B (*n* = 146) group, which was described as delinquents with early onset who were versatile with multiple risk factors, but more extreme than the Low Control A group. Finally, the last group was referred to as Normative Delinquents (*n* = 130), described as youth with few risk factors, but had pro-criminal peers, engaged in substance use, and were promiscuous (see Brennan et al., 2008 for a complete list of variables for each typology). Notably, 43% (*n* = 677) of the sample was deemed unclassifiable.

Greiner (2015) is the only known multi-wave, longitudinal treatment typology study that included a large amount of risk/need factors. Using a sample of 1,354 serious justice-involved youth (184 girls and 1,170 boys) from Philadelphia and Phoenix, LCA and Latent Transition Analysis (LTA) were used to examine typological structure and typology stability over time. Using four timepoints (baseline, 12-month follow-up, 24-month follow-up, and 36-month follow-up), results indicate that youth can be classified into three typologies: A Minimal-Needs class—with few needs across all domains, a Comprehensive-Needs class—with high needs across all domains except internalizing mental health deficits, and a Complex-Comprehensive-Needs class—scoring high on needs across all domains. Over time, the profiles remained stable; however, at the 36-month follow-up an additional class emerged (Minimal Needs—Substance using class)—characterized by elevated alcohol use and moderate antisocial personality, suggesting that profiles increase in heterogeneity over time. This research was conducted on youth; whether these results are generalizable to justice-involved adults is unknown. Further, including both genders may result in failure to identify unique effects.

Although there have been studies focusing on creating typologies of adults involved in the criminal justice system, most have focused on specific sub-populations, such as those with psychopathic traits (e.g., Swogger & Kosson, 2007) and sex offenders (e.g., Wojcik & Fisher, 2019). One of the only studies to focus on the typologies of justice involved adults more generally was Perkins (2010). This study included 733 women and 726 men who were incarcerated in a Canadian federal penitentiary and was one of the few studies to include a wide range of risk *and* strength factors. Using LCA, they found that four classes emerged for women and two classes emerged for men. Women were classified as: (1) the Potential Economic and Other class; (2) the Problematic Coping, Substances, and Associates class; (3) the Poor Mental Health and Coping class; and (4) the Overall High Need class. In contrast, men were divided into two classes: (1) the Potential Economic and Other class, and (2) the Problematic Coping, Substances, and Associates class, which were parallel to the first two classes of women. The Potential Economic and Other class included 41% of men and is described as having: a stable accommodation, an education, good coping skills, positive relationships with parents, and no history of mental health concerns. In contrast, the Problematic Coping, Substances, and Associates class included 59% of men and is described as having: substance abuse issues, peers who abuse substances, and poor coping skills in times of stressful situations. This was the only study to use an adult sample to examine typologies using a variety of risk/need and strength variables. To further advance theoretical integration and inform treatment and case management planning efforts, more research using longitudinal, multi-wave designs are required.

## Purpose of the Current Study

This body of research has typically relied on samples of justice-involved youth, have only looked at a small subset of risk factors at one timepoint and have refrained from examining strength factors in typology construction. More research is needed on the risk and strength typologies of justice-involved adults that incorporates both gender-responsive factors (e.g., mental health, adverse childhood experiences) and criminogenic needs, and includes an examination of how they may change over time. This study focuses solely on typology development to help inform the treatment and rehabilitation needs of men on community supervision. The current study assesses whether there are changes in typological membership over time, providing further understanding of how dynamic risk and strength factors may change over time. It is hypothesized that at least three unique typologies made up of a combination of risks and strengths will emerge consistently at each time. Given that there has been limited research assessing how typological membership changes over time, no hypotheses were made with respect to the stability of typological membership. It is expected that typologies with more risk factors, and especially aggression, would be more likely to reoffend.

## Method

### Participants

The sample consisted of men who initially started community supervision in Alberta, Canada between 2009 and 2012 serving a provincial community sentence.^1^ This included either stand-alone community supervision, or supervision post-release from a provincial correctional facility. The sample consisted of men assessed at three timepoints over a 9- to 14-month period (depending on time of the third assessment). The initial assessment (Time 1) had to occur within 90 days of start of supervision, Time 2 occurred 3 to 8 months post initial assessment, and Time 3 occurred 9 to 14 months post initial assessment. If an individual had more than one assessment within a time period, a random assessment was selected to represent that point in time. These specific time periods allowed for the largest sample inclusion. Those who recidivated prior to having three completed assessments were removed from analyses (to assess transitions between profiles, data is required on all timepoints for all cases).^2^ The final sample consisted of 6,675 men with an average age of 34.4 years old. About 13.0% self-identified as Indigenous.

### Measures

#### The service planning instrument (SPIn)

The SPIn (Orbis Partners, 2003) is a risk, need, and strength assessment and case management planning instrument used with adults in both institutional and community-based justice settings. Information obtained from semi-structured interviews and file-reviews are used to score the Pre-Screen version and/or the Full Assessment version of the SPIn. The full SPIn assessment contains 90 items, of which 35 are used to calculate the Pre-Screen risk and strength scores. The 90 items from the Full Assessment, make up 11 domains: Criminal history, response to supervision (e.g., institutional misconducts, violations), aggression, substance use, social influences, family, employment and education, attitudes, social and cognitive skills, stability, and mental health. Most domains contain both static and dynamic items with the exception of criminal history and response to supervision which are comprised entirely of static items. In contrast, social influences, attitudes, and social/cognitive skills are comprised entirely of dynamic items. Most domains include the assessment of both strength and risk items—however, criminal history, response to supervision, the mental health flag, and substance use domains do not contain any strength items. The Pre-Screen SPIn has predicted well across various outcomes in both community and custody samples of men and women, with AUCs ranging from 0.64 to 0.87, however, the domain scores have evidenced lower AUCs, which range from 0.54 to 0.76 (Jones & Robinson, 2018).

Specific SPIn domains will be used in the current study and are described as a function of their role in the analysis. Indicator variables are variables that are deemed endogenous to latent profiles—that is, they are utilized for typology formation. Covariates, in contrast, are considered exogenous to the model—used to predict profile membership and improve classification accuracy. Finally, auxiliary variables, not used directly in the analysis model, are examined after typological classifications to test the equality of proportions.

#### SPIn-derive indicators (18 variables)

##### Criminal history—static risk domain

This domain consists of six items assessing past offenses including youth dispositions, previous adult convictions, age at first arrest, and past incarcerations (range from 0 to 20; α = .76). Scores from 1 to 3 indicate low risk, scores of 4 to 8 indicate moderate risk, and scores of 10 or more indicate high risk.

##### Aggression/violence—dynamic risk domain and dynamic strength domain

The risk domain consists of four items assessing factors relating to violent convictions, and beliefs that put an individual at higher risk for reoffending, including opinions on verbal and physical aggression and frequency of conflicts (range from 0 to 8; α = .86). Scores of 0 to 1 indicate none to low risk, scores of 2 to 3 indicate moderate risk, and scores of 4 or more indicate high risk. The strength domain consists of four items assessing opinions and beliefs about threatening behavior that would decrease risk of reoffending (range from 0 to 8; α = .88). Scores of 1 or 2 indicate low strength, 3 or 4 indicate moderate strength, and 5 or more indicate high strength.

##### Substance use—dynamic risk domain

This domain assesses the types and number of times using various drugs and alcohol and whether it disrupts functioning. This domain assesses the use of 11 different substances including: alcohol, marijuana, cocaine/crack, ecstasy or other club drugs, heroin, hallucinogens, inhalants, amphetamines, methamphetamines, prescription drug misuse, and any other substances causing disruptions in that person’s life. Total scores range from 0 to 28, where scores from 1 to 4 indicate low risk, scores of 5 to 17 indicate moderate risk, and scores of 18 or more indicate high risk. This scale is made up of three main items, with sub-items for each substance, making it difficult to assess internal consistency.

##### Social influences—dynamic risk domain and dynamic strength domain

The risk domain consists of six items that assess antisocial peers and community engagement, and negative influences and gangs that put a person at risk for future criminal behavior (range from 0 to 26; α = .61). Scores of 1 or 2 indicate low risk, scores of 3 to 6 indicate moderate risk, and scores of 7 or more indicate high risk. The strength domain consists of five items that assess positive social activity and community engagement, and prosocial peer relationships that may act as a support (range from 0 to 15; α = .56). Scores of 1 to 4 indicating low strength, scores of 5 to 8 indicating moderate strength, and scores of 9 or more indicating high strength.

##### Family—dynamic risk domain and dynamic strength domain

The risk domain consists of seven items that assess negative family and intimate relationships, as well as marital factors, and parental factors that may increase risk of criminal behavior (range from 0 to 26; α = .46). Scores of 1 and 2 indicate low risk, scores of 3 to 7 indicate moderate risk, and scores of 8 or more indicate high risk. The strength domain consists of seven items that assess positive family and marital relationships, as well as pro-social models, attachment to children and family involvement (range from 0 to 14; α = .65). Scores of 1 to 3 indicate low strength, scores of 4 and 5 indicate moderate strength, and scores of 6 or more indicate high strength.

##### Employment—dynamic risk domain and dynamic strength domain

The risk domain consists of six items that assess employment performance, plans, and job search skills (range from 0 to 14; α = .76). Scores of 1 or 2 indicate low risk, 3 to 6 indicate moderate risk, and scores of 7 or more indicate high risk. The strength domain includes five items that assess an individual’s marketability, education, and job search skills which may assist in reducing one’s likelihood of reoffending (range from 0 to 12; α = .76). Scores of 1 or 2 indicate low strength, scores of 3 to 7 indicate moderate strength, and scores of 8 or more indicate high strength.

##### Attitudes—dynamic risk domain and dynamic strength domain

The risk domain consists of nine items that assess attitudes toward crime and the criminal justice system, and commitment to criminal lifestyle (range from 0 to 14; α = .82). Scores of 1 or 2 indicate low risk, scores of 3 to 5 indicate moderate risk, and scores of 6 or more indicate high risk. The strength domain consists of nine items that assess law-abiding attitudes, ability to accept responsibility, and willingness to make amends (range from 0 to 14; α = .87). Scores of 1 to 5 indicate low strength, scores of 6 to 10 indicate moderate strength, and scores of 11 or more indicate high strength.

##### Social/cognitive skills—dynamic risk domain and dynamic strength domain

The risk domain consists of eight items that assess hostility, impulsivity, and poor problem solving (range from 0 to 18; α = .86). Scores of 1 or 2 indicate low risk, scores of 3 and 4 indicate moderate risk, and scores of 5 or more indicate high risk. The strength domain consists of eight items that assess problem solving skills, goal setting, behavioral control, and interpersonal skills (range from 0 to 18; α = .87). Scores from 1 to 3 indicate low strength, scores of 4 to 9 indicate moderate strength, and scores of 10 or more indicate high strength.

##### Stability—dynamic risk domain and dynamic strength domain

The risk domain consists of four items that assess financial, accommodation, and transportation concerns (range from 0 to 13; α = .51). Scores of 1 or 2 indicate low risk, 3 to 5 indicate moderate risk, and scores of 6 or more indicate high risk. In contrast, the strength domain consists of four items that assess life skills, financial situation, and accommodations (range from 0 to 7; α = .48). Scores of 1 to 3 indicate low strength, 4 indicates moderate strength, and 5 or more indicates high strength.

##### Mental health flag

This is a count variable of mental health concerns, aggregated into a variable rated from 0 (*no flags*) to 2 (*two or more flags*). This variable assesses history of mental health conditions such as suicidal ideation, sexual aggression, victimization, and self-injurious behaviors. Due to the limited number of items, internal consistency could not be examined.

##### Adverse childhood experiences (ACEs)

The original ACEs study conducted by Felitti et al. (1998) found that having a greater number of 10 key negative childhood experiences (scored 0 = absent; 1 = present) increases the likelihood of problems with alcoholism, drug abuse, depression, and suicide attempts. A proxy ACE score was calculated from the SPIn Full Assessment using the following items: uses substance use to cope with trauma, comes from a single parent home, experienced physical abuse, experienced sexual abuse, experienced violence in the home, experienced instability in the home or foster care, parental substance use, and parental mental health issues. A score of 1 was added for each present item, with total scores ranging from 0 to 8 (α = .64). This method has demonstrated validity (Baglivio et al., 2015).

#### Covariate—total static risk score and age

Covariates are variables that are thought to influence responses on the indicator variables used to create the profiles. Total static risk score obtained from the SPIn Full Assessment and age were included to examine any differences resulting specifically from individuals’ static risk score or age. Static factors are unchangeable factors, such as historical information (e.g., response to supervision, history of homelessness). Total static risk scores ranging from 1 to 20 are considered low, scores ranging from 21 to 47 are considered moderate, and scores of 48 or more are considered high. Internal consistency was found to be good (α = .84). Age at time of initial SPIn assessment was used as the second covariate to examine any differences that would result as a product of individuals’ biological age. The age ranged from 16 to 83 (*M* = 34.4, *SD* = 11.6) at the start of supervision.

#### Auxiliary variable—Indigenous status

Auxiliary variables are not used directly in the analysis model but are examined after the LPAs are conducted to examine differences in proportions in the composition of the typologies. Indigenous status was used post-analysis, to assess the extent to which profile membership varied among non-Indigenous and Indigenous men (i.e., First Nations, Metis, or Inuit). Indigenous status was a dichotomous variable, used to indicate if the individual self-identified as Indigenous (yes/no).

#### Distal outcomes

There were three dichotomous (yes/no) distal outcomes of interest that were examined independently. Each of these outcomes were measures of reoffending that were based on re-offense records where there was recontact with correctional services in the province of Alberta. The outcomes were: (1) Any new charge(s), which includes new charges that are non-violent, sexual, or violent in nature, but excludes any technical violations; (2) Any new violent charge(s), which includes crimes against the person that range in severity from threats of harm to death. Specifically, this includes uttering threats, assault (including causing bodily harm, assault with a weapon, assault of a peace officer, and simple assaults), any weapon-related offenses (including pointing a firearm, possession, and careless storage), harassment, robbery, dangerous driving/operation causing bodily harm, and any murder charges (but not sexual-based charges); and (3) technical violation(s), which includes any breaches of court-ordered or community supervision conditions resulting in a failure to comply, or failure to appear. Each outcome was assessed over a 3-year fixed follow-up from the time of initial SPIn Full Assessment, which translates to 22 to 27 months post Time 3, as Time 3 assessments occurred between 9 and 14 months post initial assessment.

### Analyses

Instead of focusing on the relationships among *variables*, these models focus specifically on the behavior of *individuals*. That is, Latent Profile Analysis (LPA), unlike factor analysis, classifies individuals into various typologies based on comparable patterns of individual characteristics. Latent Transition Analysis (LTA) examines changes in profiles with longitudinal data, assessing transitional probabilities, to inform the probability of transitioning between profiles at different times (Collins & Lanza, 2010).

To determine the relative model fit across the various numbers of profiles, several fit indices criteria were used, including Akaike’s Information Criterion (AIC), Bayesian Information Criterion (BIC), and sample-size adjusted Bayesian Information Criterion (ABIC); lower values on each indicated better model fit. Entropy was examined and values closer to 1 indicated better model fit. Lo-Mendell-Rubin test was also used to determine whether a *k* profile model fit the data better than a *k* − 1 profile model. The best fitting model was determined based on fit indices criteria, theory, and through interpretation of the various profile structures.

First, LPAs were conducted at Time 1 and fit indices were used to determine the number of profiles that best fit the data. Second, LPAs for Time 2 and Time 3 were conducted to examine the number of profiles that emerged at these later timepoints. Third, age, total static risk score, and Indigenous status were examined at each timepoint. Next, latent transition probabilities were examined between each timepoint to assess changes in profile membership over time. Notably, latent transitions can only be examined if similar profiles emerged at each timepoint. Finally, the relationship between latent transitions and three distal outcomes (technical violations, any new charges, and violent charges) were examined.

## Results

### Sample Descriptives

Just over half of the men were low static risk (55.1%), about 38.6% were moderate static risk, and 6.4% were high static risk. In terms of index offenses, 23.4% committed a non-violent offense, 42.8% committed a violent offense, and 4.6% committed a sexual offense. Based on the initial SPIn, the average total dynamic risk score was 22.3 (*SD* = 16.9) and the average total dynamic strength score was 28.7 (*SD* = 16.7). In terms of reoffending outcomes, 10.7% were charged with any new offense, 6.2% were charged with a violent offense, and 6.3% had a technical violation 3-years post initial SPIn assessment.

### Data Screening

Because data was aggregated into three time periods, there were no missing data across the timepoints for any of the 18 indicators, covariates, and auxiliary variables. The covariance coverage for all three timepoints indicated good coverage. The majority of domain scores across all timepoints were positively skewed. However, given that it is expected that latent profile models are made up of a variety of normal distributions from different groups of individuals, variables are treated as normally distributed (Kreuter & Muthén, 2008).

### LPA Results at Time 1

A 2- to 6-profile model solution were run sequentially to identify the best fitting model (see Table 1). Upon considering model fit indices and theory, it was determined that a 5-structure profile solution fit best. Posterior profile membership probabilities were also examined at each timepoint and ranged from 0.90 to 0.98, which is considered good.

**Table 1.** Relative Fit Statistics for Time 1, 2, and 3.

Profile	AIC	BIC	ABIC	Entropy	LMR	*p*
Time 1						
2-Structure	541,479.42	541,853.76	541,678.98	.901	16,540.76	<.001
3-Structure	528,938.44	529,442.09	529,206.94	.920	12,504.25	.029
4-Structure	522,340.77	522,973.74	522,678.21	.940	6,596.24	.015
**5-Structure**	**517,377.30**	**518,139.58**	**517,783.67**	**.907**	**4,971.76**	<**.001**
6-Structure	514,070.71	514,962.32	514,546.03	.905	3,324.71	.146
Time 2						
2-Structure	537,567.53	537,941.86	537,767.09	.898	16,832.69	<.001
3-Structure	525,696.94	526,200.59	525,965.44	.919	11,837.84	<.001
4-Structure	519,253.61	519,886.58	519,591.05	.936	6,442.83	<.001
5-Structure	514,113.91	514,876.20	514,520.29	.906	5,146.93	<.001
6-Structure	510,588.58	511,480.18	511,063.89	.905	3,542.16	.007
Time 3						
2-Structure	534,786.27	535,160.61	534,985.83	.895	16,834.04	<.001
3-Structure	523,015.19	523,518.84	523,283.69	.919	11,738.93	.017
4-Structure	516,574.57	517,207.54	516,912.01	.937	6,440.13	.045
5-Structure	511,503.16	512,265.45	511,909.54	.904	5,079.05	<.001
6-Structure	507,986.36	508,877.97	508,461.68	.905	3,533.68	<.001
7-Structure	507,355.14	508,376.06	507,899.40	.915	3,251.99	.125

*Note.* AIC = Akaike’s Information Criterion; BIC = Bayesian Information Criteria; ABIC = Sample size adjusted Bayesian Information Criteria; LMR = Lo-Mendell-Rubin test.

The bold values represent the profile structure that best fit the data (and was selected) for each time point.

#### Profile 1: Low risk/Low strength

This profile is defined as scoring the lowest on all dynamic risk scores, criminal history, mental health, and adversity, relative to all other profiles. This profile also scores low on all dynamic strength domains relative to most other profiles, and particularly, scores the lowest on the family strength domain in comparison to all other profiles.

#### Profile 2: Aggressive, complex need/Low strength

This profile is defined as having complex need because of high scores on both traditional criminogenic needs and non-criminogenic needs (i.e., mental health and adverse childhood experiences) relative to the other profiles—apart from profile 5. Specifically, this profile scores highest on the skills risk, attitudes risk, and family risk domains. Men in this profile score substantially higher than the remaining profiles on aggression. Finally, this profile scores low on all strength domains, and in particular, scores the lowest on the dynamic aggression strength domain.

#### Profile 3: Moderate risk/Moderate strength

Relative to the other profiles, this profile is defined as scoring moderately on all domains. That is, this profile does not score the highest nor the lowest on any of the domains including any dynamic risk and strengths domains, the mental health domain, adversity, or criminal history.

#### Profile 4: Low risk/High strength

Similar to the Low risk/Low strength profile, this profile scores lowest on all risk domains, as well as mental health and adversity. Although, compared to the Low risk/Low strength domain, this profile has slightly higher scores on criminal history, social risk, drug use, stability risk, mental health, and adversity. This profile, however, scores highest on all strength domains—scoring substantially higher than any other profile. Notably, the highest strength score is on the dynamic skills strength domain.

#### Profile 5: Non-aggressive, complex need/Low strength

**Figure 1.**
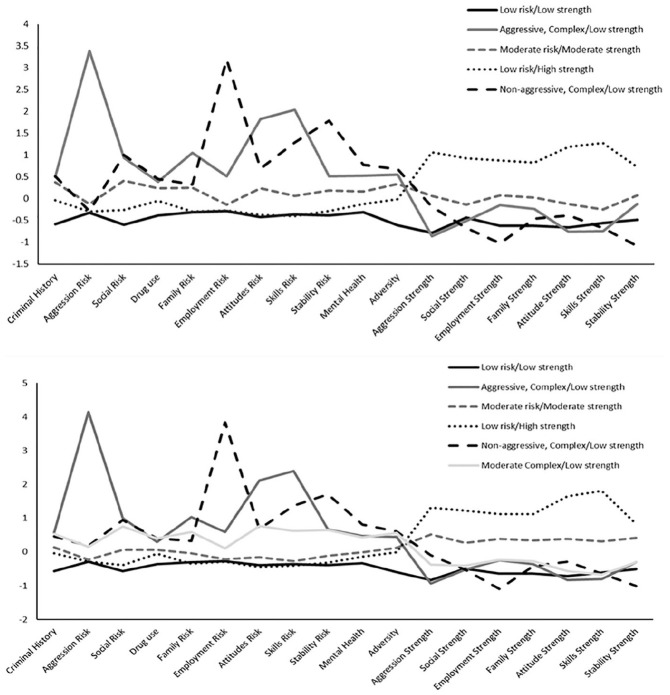
Comparison of standardized domain scores for profiles at Time 1 and Time 3. *Note.* The same five profiles emerged at Time 1 and Time 2, whereas at Time 3, six profiles emerged.

This profile scores highest on many of the dynamic risk domains in comparison to all other profiles. This profile scores substantially higher on the dynamic employment risk and stability risk domains than any of the other profiles, indicating that these men have issues with employment and securing finances, accommodation, and transportation. Relative to all other profiles, men in this profile score highest on the mental health flag and adversity. This profile scores just as low on the dynamic aggression risk domain as the two low risk profiles (Low risk/Low strength and Low risk/High strength profiles), and relative to all other profiles, scores lowest on the dynamic employment strength and stability strength domains. The estimated means and standard deviations for each profile is in Table 2. The standardized risk and strength scores for each profile are in Figure 1.

**Table 2.** Means for Each of the Risk and Strength Domains across the Five Profiles at Time 1.

Variables in LPA	Profile 1 (*n* = 1,952)		Profile 2 (*n* = 408)		Profile 3 (*n* = 2,244)		Profile 4 (*n* = 1,718)		Profile 5 (*n* = 353)	
	*M*	*SD*	*M*	*SD*	*M*	*SD*	*M*	*SD*	*M*	*SD*
Criminal history	2.87	(3.07)	8.73	(5.75)	8.13	(5.36)	5.90	(5.16)	8.88	(5.62)
Aggression^a^—Risk	0.01	(0.13)	3.87	(1.39)	0.22	(0.52)	0.04	(0.24)	0.54	(0.85)
Aggression—Strength	0.23	(0.82)	0.14	(0.42)	2.01	(1.60)	4.04	(1.63)	1.50	(1.60)
Substance use—Risk	3.17	(4.54)	8.99	(8.87)	7.89	(7.53)	5.74	(7.15)	9.59	(11.97)
Social influence—Risk	0.66	(1.32)	5.54	(4.52)	3.95	(3.01)	1.77	(2.10)	5.72	(3.99)
Social influence—Strength	3.18	(2.10)	2.96	(2.87)	4.20	(2.84)	7.81	(3.23)	2.44	(2.43)
Family—Risk	3.13	(3.07)	8.85	(5.24)	5.50	(4.35)	3.23	(3.36)	5.81	(4.50)
Family—Strength	1.88	(1.86)	3.00	(2.17)	3.72	(2.27)	5.95	(2.88)	2.33	(1.94)
Employ—Risk	0.12	(0.46)	1.38	(2.21)	0.36	(0.76)	0.14	(0.57)	5.58	(2.57)
Employ—Strength	1.66	(1.16)	3.16	(2.81)	3.80	(2.64)	6.23	(3.05)	0.53	(0.91)
Attitudes—Risk	0.30	(0.68)	5.20	(3.83)	1.77	(2.00)	0.42	(0.98)	2.73	(2.72)
Attitudes—Strength	2.57	(1.60)	2.19	(2.62)	4.90	(3.09)	10.68	(3.70)	3.85	(3.53)
Skills^b^—Risk	0.13	(0.55)	4.79	(3.45)	1.01	(1.60)	0.10	(0.40)	3.34	(2.83)
Skills—Strength	1.63	(1.35)	0.93	(1.61)	2.80	(2.48)	8.66	(3.36)	1.22	(1.81)
Stability—Risk	0.54	(1.05)	2.19	(2.24)	1.62	(1.81)	0.76	(1.21)	4.51	(2.45)
Stability—Strength	3.24	(1.07)	3.87	(1.92)	4.18	(1.55)	5.25	(1.32)	2.29	(1.48)
Mental health—Flag	0.44	(0.75)	1.21	(0.92)	0.87	(0.92)	0.63	(0.85)	1.43	(0.84)
ACEs^c^	0.09	(0.33)	1.65	(2.61)	1.36	(1.41)	0.91	(1.24)	1.85	(1.61)

*Note.* Profile 1 = Low risk/low strength, Profile 2 = Aggressive, complex need/low strength, Profile 3 = Moderate risk/moderate strength, Profile 4 = Low risk/high strength, and Profile 5 = Non-aggressive, complex need/low strength. *M* = mean; *SD* = standard deviation.

a Aggression refers to the aggression/violence domain.

b Skills refers to the cognitive/social skills domain.

c ACEs refers to adverse childhood experiences.

### LPA Time 2 and Time 3

At Time 2, it was determined that a 5-structure profile solution fit the data best (see Table 1), and probabilities of correct classification for the five profiles ranged from 0.90 to 0.99. Although the means and standard deviations changed slightly, the same trends and profiles emerged at Time 1 and Time 2 (as such, see profile descriptions for Time 1). At Time 3 it was determined that a 6-structure profile solution fit the data best (see Table 1). The probabilities of correct classification for the six profiles ranged from 0.89 to 0.99. While the same five profiles emerged at each timepoint, at Time 3, a new sixth profile of men emerged.

#### Profile 6: Moderate complex need/Low strength

Relative to the other profiles, this profile scores moderately across all dynamic risk domains, as well as the mental health flag. That is, this profile does not score the highest or the lowest on mental health or dynamic risk domains. This profile scores similarly on the adversity to Profile 2 (Aggressive, complex need/Low strength) and Profile 5 (Non-aggressive, complex need/Low strength). Similar to most other profiles other than Profile 2 (Aggressive, complex need/Low strength), this profile is non-aggressive and scores low on dynamic strength domains. The estimated means and standard deviations for each of the profiles are presented in Table 3. See Figure 1 for the standardized risk and strength scores for the six profiles at Time 3.

**Table 3.** Means for Each of the Risk and Strength Domains across the Six Profiles at Time 3.

Variables in LPA	Profile 1 (*n* = 1,977)		Profile 2 (*n* = 268)		Profile 3 (*n* = 2,246)		Profile 4 (*n* = 875)		Profile 5 (*n* = 260)		Profile 6 (*n* = 1,049)	
	*M*	*SD*	*M*	*SD*	*M*	*SD*	*M*	*SD*	*M*	*SD*	*M*	*SD*
Criminal history	3.15	(3.34)	9.19	(5.89)	6.88	(5.25)	5.96	(5.35)	8.60	(5.55)	9.03	(5.40)
Aggression^a^—Risk	0.01	(0.10)	3.90	(1.32)	0.06	(0.29)	0.01	(0.12)	0.42	(0.79)	0.40	(0.67)
Aggression—Strength	0.33	(0.93)	0.13	(0.40)	3.16	(1.54)	4.79	(1.60)	1.87	(1.67)	1.30	(1.40)
Substances—Risk	2.81	(4.19)	6.96	(6.71)	5.52	(6.03)	4.77	(6.14)	7.62	(10.26)	7.57	(7.10)
Social^b^—Risk	0.76	(1.45)	5.38	(4.49)	2.62	(2.40)	1.25	(1.78)	5.23	(4.14)	4.64	(3.42)
Social—Strength	3.24	(2.12)	3.14	(3.18)	5.85	(3.12)	9.08	(3.03)	3.14	(3.04)	3.55	(2.68)
Family—Risk	3.17	(3.02)	8.47	(5.02)	4.19	(3.66)	2.95	(3.17)	5.65	(4.74)	6.70	(4.53)
Family—Strength	2.03	(1.93)	2.83	(2.13)	4.83	(2.46)	7.11	(3.18)	2.64	(2.07)	3.09	(2.00)
Employ—Risk	0.14	(0.48)	1.43	(2.09)	0.22	(0.65)	0.13	(0.52)	6.26	(2.34)	0.72	(1.04)
Employ—Strength	1.85	(1.40)	3.10	(2.64)	5.07	(2.77)	7.36	(3.22)	0.47	(0.83)	3.17	(2.54)
Attitudes—Risk	0.33	(0.76)	5.40	(3.95)	0.80	(1.33)	0.22	(0.62)	2.50	(2.52)	2.66	(2.38)
Attitudes—Strength	2.70	(1.72)	2.17	(2.77)	7.63	(3.19)	13.33	(2.99)	4.58	(3.83)	3.37	(2.70)
Skills^c^—Risk	0.12	(0.45)	5.02	(3.55)	0.26	(0.70)	0.03	(0.19)	3.19	(2.94)	1.87	(2.09)
Skills—Strength	1.71	(1.38)	0.94	(1.67)	5.46	(2.81)	11.42	(2.84)	1.61	(2.15)	1.50	(1.79)
Stability—Risk	0.51	(1.02)	2.34	(2.44)	0.99	(1.36)	0.63	(1.09)	4.15	(2.49)	2.33	(2.08)
Stability—Strength	3.40	(1.13)	3.73	(1.98)	4.90	(1.38)	5.56	(1.18)	2.59	(1.51)	3.72	(1.66)
Mental health	0.44	(0.75)	1.15	(0.92)	0.72	(0.88)	0.61	(0.85)	1.45	(0.83)	1.12	(0.93)
ACEs^d^	0.14	(0.45)	1.51	(1.53)	1.09	(1.32)	0.91	(1.25)	1.73	(1.58)	1.67	(1.57)

*Note*. Profile 1 = Low risk/Low strength, Profile 2 = Aggressive, complex need/Low strength, Profile 3 = Moderate risk/Moderate strength, Profile 4 = Low risk/High strength, Profile 5 = Non-aggressive, complex need/Low strength, and Profile 6 = Moderate complex need/Low strength. *M* = mean; *SD* = standard deviation.

a Aggression refers to the aggression/violence domain.

b Social refers to social influences domain.

c Skills refers to the cognitive/social skills domain.

d ACEs refers to adverse childhood experiences.

### Covariate Analyses

There were no significant differences in age among the profiles at each timepoint, indicating that age did not inform typology formation (see Table 4). Total static risk scores were significantly different across most pairs of profiles, with a few exceptions. At Time 1 the Aggressive, complex need/Low strength profile and the Non-aggressive, complex need/Low strength profile did not significantly differ in terms of total static risk scores (40.3 vs. 38.2). At Time 2, total static risk scores for the Moderate risk/Moderate strength profile and the Low risk/High strength profile did not significantly differ (28.2 vs. 20.5). At Time 3, the Non-aggressive, complex need/Low strength profile and the Moderate complex need/Low strength profile had static risk scores that were similar (36.7 vs. 35.1). Across each timepoint, the Aggressive, complex need/Low strength had the highest total static risk score and the Low risk/Low strength profile had the lowest total static risk score (see Table 4).

**Table 4.** Mean Age and Static Risk Score for Each Profile across Timepoints.

Profile	Time 1		Time 2		Time 3	
	*M*	*SD*	*M*	*SD*	*M*	*SD*
Age						
Profile 1	33.84	(11.99)	33.82	(12.03)	33.79	(11.94)
Profile 2	34.94	(10.67)	34.97	(10.64)	35.36	(11.22)
Profile 3	34.62	(11.34)	34.82	(11.35)	34.39	(11.31)
Profile 4	34.66	(11.42)	34.37	(11.26)	34.77	(11.18)
Profile 5	33.99	(12.77)	34.10	(13.18)	34.52	(13.49)
Profile 6	—	—	—	—	34.90	(11.46)
Static risk score						
Profile 1	10.25	(6.99)	9.92	(6.97)	10.92	(8.15)
Profile 2	40.33	(19.72)	40.15	(19.88)	41.50	(20.16)
Profile 3	29.41	(15.92)	28.21	(15.80)	23.23	(14.53)
Profile 4	19.55	(14.22)	20.48	(15.03)	19.13	(14.93)
Profile 5	38.23	(18.64)	36.30	(18.08)	36.73	(17.69)
Profile 6	—	—	—	—	35.12	(16.84)

*Note*. At Time 1 and Time 2: Profile 1 = Low risk/Low strength, Profile 2 = Aggressive, complex need/Low strength, Profile 3 = Moderate risk/Moderate strength, Profile 4 = Low risk/High strength, and Profile 5 = Non-aggressive, complex need/Low strength. At Time 3, Profiles 1 to 5 were the same as Time 2; however, a new profile emerged: Profile 6 = Moderate complex need/Low strength. *M* = mean; *SD* *=* standard deviation.

### Auxiliary Analyses

There were significant differences between profiles in terms of the proportions of Indigenous and non-Indigenous men across all timepoints. At Time 1, almost half of all Indigenous men (45.9%) were in the Moderate risk/Moderate strength profile, whereas only 31.8% of all non-Indigenous men were in this profile (χ^2^ = 67.41, *p* < .001). A higher proportion of non-Indigenous men were in the Low risk/Low strength profile than Indigenous men (31.5% vs. 14.3%; χ^2^ = 107.50, *p* < .001; see Table 5). At Time 2, almost half of all Indigenous men (44.0%) were in the Moderate risk/Moderate strength profile, whereas 32.5% of all non-Indigenous men were in this profile (χ^2^ = 43.70, *p* < .001). At Time 3, 38.5% of Indigenous men were in the Moderate risk/Moderate strength profile, whereas 32.9% of all non-Indigenous men were in this profile (χ^2^ = 10.61, *p* < .001). The magnitude of the difference between these profiles decreased from Time 1 to Time 3.

**Table 5.** Time 1, 2, and 3 Proportions of Indigenous and Non-Indigenous Men in Each Profile.

Profile	Percentage of Indigenous men	Percentage of non-Indigenous men	χ^2^	*p*
Time 1				
Profile 1	14.30	31.47	107.50	<.001
Profile 2	5.65	6.19	0.37	.544
Profile 3	45.91	31.78	67.41	<.001
Profile 4	24.80	25.88	0.46	.497
Profile 5	9.34	4.68	32.70	<.001
Time 2				
Profile 1	13.84	29.89	96.80	<.001
Profile 2	5.19	4.94	0.10	.753
Profile 3	43.95	32.54	43.70	<.001
Profile 4	27.68	27.60	0.01	.960
Profile 5	9.34	5.03	26.62	<.001
Time 3				
Profile 1	15.46	31.74	95.87	<.001
Profile 2	4.04	4.01	0.01	.998
Profile 3	38.52	32.92	10.61	.001
Profile 4	12.11	13.26	0.87	.351
Profile 5	6.92	3.44	24.36	<.001
Profile 6	22.95	14.63	39.40	<.001

*Note.* Time 1 and 2: Profile 1 = Low risk/Low strength, Profile 2 = Aggressive, complex need/Low strength, Profile 3 = Moderate risk/Moderate strength, Profile 4 = Low risk/High strength, and Profile 5 = Non-aggressive, complex need/Low strength. Time 3: Profile 1 = Low risk/Low strength, Profile 2 = Aggressive, complex need/Low strength, Profile 3 = Moderate risk/Moderate strength, Profile 4 = Low risk/High strength, Profile 5 = Non-aggressive, complex need/Low strength, and Profile 6 = Moderate complex need/Low strength. χ^2^ = Chi-square test.

### Latent Transitional Probabilities

Transitional probabilities could not be calculated between Time 2 and Time 3, as the interpretability of the results would not be meaningful, given that the number of profiles that emerged in Time 3 (six profiles) was different from Time 2 (five profiles). Nonetheless, transitional probabilities were examined between Time 1 and Time 2. Across these two timepoints, most individuals remained in the same profile (see Table 6 for profile counts and proportions). The largest amount of movement was from the Aggressive, complex need/Low strength profile (at Time 1) to the Moderate risk/Moderate strength profile (at Time 2), although this represented only 1.2% of the sample (*n* = 78). Overall, only 5.6% of the sample switched to a different profile from Time 1 to Time 2 (*n* = 372).

**Table 6.** Profile Transitions from Time 1 to Time 2.

Profile transitions	(*N* = 6,675)	
	Count	%
**Profile 1 to Profile 1**	1,859	27.84
Profile 1 to Profile 2	3	0.05
Profile 1 to Profile 3	17	0.26
Profile 1 to Profile 4	24	0.36
Profile 1 to Profile 5	8	0.12
Profile 2 to Profile 1	0	0
**Profile 2 to Profile 2**	307	4.60
Profile 2 to Profile 3	78	1.17
Profile 2 to Profile 4	7	0.11
Profile 2 to Profile 5	16	0.24
Profile 3 to Profile 1	1	0.02
Profile 3 to Profile 2	20	0.30
**Profile 3 to Profile 3**	2,095	31.38
Profile 3 to Profile 4	64	0.96
Profile 3 to Profile 5	33	0.49
Profile 4 to Profile 1	2	0.03
Profile 4 to Profile 2	2	0.03
Profile 4 to Profile 3	32	0.48
**Profile 4 to Profile 4**	1,741	26.08
Profile 4 to Profile 5	3	0.05
Profile 5 to Profile 1	0	0
Profile 5 to Profile 2	6	0.09
Profile 5 to Profile 3	49	0.73
Profile 5 to Profile 4	7	0.11
**Profile 5 to Profile 5**	301	4.50

*Note.* The transitions that are bold represent those individuals who remained in the same profile from Time 1 to Time 2 (indicating no change in profile membership). Profile 1 = Low risk/Low strength, Profile 2 = Aggressive, complex need/Low strength, Profile 3 = Moderate risk/Moderate strength, Profile 4 = Low risk/High strength, and Profile 5 = Non-aggressive, complex need/Low strength.

### Typological Structure and Criminal Outcomes

Distal outcomes were examined in relation to profile membership at Time 3. As seen in Tables 7 and 8, the Low risk/High strength profile had the lowest proportion of reoffending outcomes, although the proportions of reoffending for the Low risk/Low strength profile were similar. In contrast, the Aggressive, complex need/Low strength profile had the highest proportion of technical violations and violent offenses, and the Non-aggressive, complex need/Low strength had the highest proportion of any new offenses. However, the Aggressive, complex need/Low strength profile had similar rates of reoffending to the Non-Aggressive, complex need/Low strength profile, and the Moderate complex need/Low strength profile.

**Table 7.** Proportion of Men Who Reoffended from Each Profile at Time 3.

Profiles	TV		Any new		Violent	
	*n*	%	*n*	%	*n*	%
Profile 1	78	3.9	157	7.9	86	4.4
Profile 2	38	14.2	43	16.0	29	10.8
Profile 3	120	5.3	240	10.7	143	6.4
Profile 4	29	3.3	56	6.4	22	2.5
Profile 5	34	13.1	45	17.3	28	10.8
Profile 6	121	11.5	176	16.8	104	9.9

*Note.* TV = Technical violations. Profile 1 = Low risk/Low strength (*n* = 1,977), Profile 2 = Aggressive, complex need/Low strength (*n* = 268), Profile 3 = Moderate risk/Moderate strength (*n* = 2,246), Profile 4 = Low risk/High strength (*n* = 875), Profile 5 = Non-aggressive, complex need/Low strength (*n* = 260), and Profile 6 = Moderate complex need/Low strength (*n* = 1,049).

**Table 8.** Profile Comparisons of Criminal Outcomes at Time 3.

Profile comparisons	Technical violation		Any new charge		Violent charge	
	*OR*	*p*	*OR*	*p*	*OR*	*p*
Profile 1 to Profile 2	0.249	<.001	0.453	<.001	0.381	<.001
Profile 1 to Profile 3	0.719	.013	0.723	.001	0.679	.001
Profile 1 to Profile 4	1.117	.646	1.250	.236	1.844	.078
Profile 1 to Profile 5	0.271	<.001	0.426	<.001	0.387	<.001
Profile 1 to Profile 6	0.335	<.001	0.453	<.001	0.443	<.001
Profile 2 to Profile 3	2.891	.001	1.597	.042	1.781	.046
Profile 2 to Profile 4	4.492	.003	2.760	.004	4.837	.010
Profile 2 to Profile 5	1.091	.747	0.940	.789	1.016	.955
Profile 2 to Profile 6	1.347	.211	1.001	.997	1.162	.542
Profile 3 to Profile 4	1.554	.116	1.728	.012	2.716	.014
Profile 3 to Profile 5	0.377	<.001	0.588	<.001	0.571	.001
Profile 3 to Profile 6	0.466	<.001	0.626	<.001	0.653	<.001
Profile 4 to Profile 5	0.243	<.001	0.340	<.001	0.210	<.001
Profile 4 to Profile 6	0.300	<.001	0.363	<.001	0.240	<.001
Profile 5 to Profile 6	1.235	.377	1.065	.753	1.144	.596

*Note.* OR = Odds ratio. Profile 1 = Low risk/Low strength, Profile 2 = Aggressive, complex need/Low strength, Profile 3 = Moderate risk/Moderate strength, Profile 4 = Low risk/High strength, Profile 5 = Non-aggressive, complex need/Low strength, and Profile 6 = Moderate complex need/Low strength.

## Discussion

Trajectory-based research examines the development of criminal behavior over time (e.g., how it increases, decreases, stays the same) and focuses predominately on adolescence. That is, this research typically defines groups based on the extent of change over time. In contrast, treatment typology research tends to group individuals based on their risk factors to help inform effective correctional treatment interventions and aid in understanding how various factors may operate together. Examining typological changes over time can assist with ensuring the most appropriate and up-to-date treatment plan is provided. Unfortunately, typological research has failed to include strength factors. As such, the current study was the first to incorporate risks, needs, and strengths to assess typologies of men on community supervision, and examine how these typologies change over time. A secondary goal was to examine the relationship between typologies and reoffending outcomes, such as technical violations, new charges, and violent charges. Results demonstrated that men on community supervision can be classified into five distinct typologies based on treatment needs and strengths. The following profiles emerged at each timepoint: (1) Low risk/Low strength profile scoring low on all domains; (2) Aggressive, complex need/Low strength profile scoring high on aggression and all risk domains including mental health and ACEs, but low on strength domains; (3) Moderate risk/Moderate strength profile scoring moderate on all domains; (4) Low risk/High strength profile scoring low on all risk domains including mental health and ACEs, but highest on all strengths; and (5) Non-aggressive, complex need/Low strength profile scoring high on risk domains (employment, stability), mental health, and ACEs, but low on aggression and strengths.

It is interesting that two distinct types of low risk profiles emerged, one scoring low and one scoring high on strengths. This may have implications on the treatment and classification of those who are low risk. For instance, perhaps there are additional categories of justice-involved individuals other than low, moderate, or high risk that need to be considered when determining frequency of contact with community supervision officers, program placements, and even probation conditions. In addition, having two “types” of low risk justice-involved individuals can have implications for how these individuals are managed in the community. For example, some jurisdictions implement a low-intensity supervision model whereby those rated lowest risk report monthly to a computerized kiosk, rather than to a community supervision officer, to answer questions regarding their contact details, employment, and any problems they may be facing (Barnes et al., 2010). This approach allows for more resources to be placed toward higher risk cases and has been found to be successful in reducing the rate of re-arrests over a 2-year period (Wilson et al., 2007). It is important to examine whether higher risk samples also display similar strength patterns—as this could be a potential direction for the use of overrides (i.e., security classification, programming needs, and frequency of contact with supervision officers).

While the lower risk cases were differentiated in terms of high/low strengths, the higher risk group in the sample were also further differentiated in terms of what makes them higher risk. That is, there were two higher risk groups that emerged—both scored high on an array of needs, including mental health and ACEs, and low on strengths; however, one was characterized as aggressive and violent, whereas the other group was characterized as non-aggressive, but scored very high (relative to the other profiles) on employment risk and lifestyle stability risk. This may have implications on the treatment and classification of those who are higher risk. Specifically, among the aggressive higher risk group, treatment plans can be geared toward anger management, reducing violent tendencies, and problem solving, whereas for the non-aggressive higher risk group, treatment plans can be geared toward employability, goal setting, addressing housing and transportation concerns, and other issues related to lifestyle stability.

Another finding was that mental health and ACEs co-occurred with higher criminogenic need/risk factors. That is, among the profiles that scored highest on more traditional risk factors/criminogenic needs, the mental health, and ACE scores were also highest for these profiles. Notably, there were no profiles that emerged that scored high on ACEs and mental health, but low on traditional risk factors/criminogenic needs. This has implications for case management planning, whereby those who are high risk/need should receive treatment that not only targets their criminogenic needs but does so in a trauma-informed way. Unfortunately, there has been limited research on trauma-informed services with justice-involved men (with the exception of men sex offenders; see Janssen, 2018). However, research has found that ACEs can lead to several issues, including mental health concerns, problems maintaining relationships, and behavioral problems (Ford et al., 2012), which trauma-informed services can assist with.

### Stability of Typologies

Findings indicate the same five profiles emerged at Time 1 and Time 2, but at Time 3, a sixth profile-structure emerged, which included a Moderate complex need/Low strength profile. Overall, from Time 1 to Time 2, there was a slight increase in attitude strength scores and slight decrease in stability strength and substance use risk scores across each profile. Although decreases in substance use and increases in prosocial attitudes were expected as successful time in the community increase, it was not expected that stability strength scores would decrease. The Low risk/Low strength profile and the Aggressive, complex need/Low strength profile had similar mean scores across all domains and timepoints. The Moderate risk/Moderate strength profile, the Low risk/High strength profile, and the Non-aggressive, complex need/Low strength (profiles 3–5), while demonstrating similar *trends* in scores on each domain, had different mean scores on the majority of risk, need, and strength domains. However, given that an additional profile emerged at the third timepoint, the change in domain scores in the profiles are most likely due to the different typological structure, rather than due to men’s change in scores (especially considering the discrepancy in sample sizes in typologies from Time 1 to Time 3; for example Profile 4 at Time 1 [*n* = 1,718] and at Time 2 [*n* = 1,843] was much larger than at Time 3 [*n* = 875]). Furthermore, the study conducted by Wanamaker and Brown (2021), which included the current sample of men, indicated that overall dynamic strength scores tended to increase and overall dynamic risk scores tended to decrease over time; albeit these changes were quite minimal (see Wanamaker & Brown for more details). This further demonstrates that major changes in domain scores across profiles are likely primarily due to the different typological structures.

### Stability of Typological Membership

Transitions between profiles could only be assessed between Time 1 and Time 2 due to a different profile structure that emerged at Time 3. Overall, only 5.6% of men switched from one profile to another between Time 1 and Time 2. This limited change did not seem to follow a specific pattern. There are several plausible reasons for this. First, the sample included in the current analyses are mainly low risk (55.1% based on SPIn overall risk score) and individuals who reoffended within 9- to 14-months of the initial assessment (based on when the last SPIn assessment occurred) were excluded, limiting room for change on dynamic risk domains. Second, change in risk, need, and strength domains may require more time. Given that transitions were assessed only between Time 1 and Time 2, there are only a matter of months between these two timepoints. Time 2 assessments occur between 3- and 8-months post initial assessment, which may not be enough time to exhibit changes in the various dynamic domains.

The SPIn is comprised of dynamic items which are combined to create dynamic domains; however, it is important to consider that not all dynamic items change at the same rate. Research has found that dynamic items can be divided into two categories: stable dynamic—factors that are more long-standing that change over a matter of months or years, and acute dynamic—factors that change more rapidly, such as days or weeks (Hanson et al., 2007). The extent to which items making up the dynamic SPIn domains are stable versus acute is unknown. Thus, the limited change in typological memberships may be due to the number of items that are stable dynamic relative to the number of items that are acute dynamic. In addition, although most indicators included in the analyses were dynamic in nature, two were not—criminal history and ACEs, both of which were comprised predominately of static items. While these variables are important to consider for typology development, their static nature may further limit one’s ability to change from one profile to another over time.

### Typological Membership and Criminal Outcomes

One of the main reasons for identifying typologies is to determine if there are certain groups of justice-involved individuals that are more likely to reoffend (Jones & Harris, 1999). In turn, treatment and rehabilitation efforts can be tailored to target the domains most pertinent to those individuals and better inform supervision efforts (e.g., frequency of contact). There were three profiles that had the highest rates of criminal outcomes: the Aggressive, complex need/Low strength profile, the Non-aggressive, complex need/Low strength profile, and the Moderate complex need/Low strength profile. An interesting finding was that those who were non-aggressive had similar rates of reoffending, including violent outcomes, to those who were aggressive, as long as they scored high on complex needs (dynamic risks as well as mental health needs and ACEs). As such, men who have complex needs tend to be more likely to reoffend than men who score moderately on criminogenic risk/need factors alone (e.g., Moderate risk/Moderate strength). Conversely, all outcomes were lowest for those who scored low on risk domains, regardless of strength scores (e.g., Low risk/Low strength and Low risk/High strength).

### Indigenous Men and Typological Membership

There were significant differences in the proportion of Indigenous and non-Indigenous men making up the profiles. A larger proportion of Indigenous men made up profiles characterized by more strengths (e.g., Moderate risk/Moderate strength, and Low risk/High strength profiles). Although the reason for this finding is unclear and requires further investigation, results highlight that there are differences between Indigenous and non-Indigenous men that need to be considered. It is not enough to classify men into various typologies—other factors must be considered, including ethnicity and social economic status. Identifying whether unique typologies emerge among Indigenous men, incorporating culturally-relevant risk, need, and strength factors, can assist with tailoring treatment to target appropriate need domains.

### Limitations and Directions for Future Research

Several limitations emerged as a function of utilizing administrative data. First, ensuring that there were three assessment periods resulted in losing cases who recidivated within the 14-month period and limiting the sample to those who are predominately lower risk. Although this may affect the generalizability to all men on community supervision, supplementary research reported in Wanamaker (2020) demonstrates a similar typological structure that emerged among all men with an initial SPIn Full Assessment within 90 days of start of community supervision. In addition, given the timepoint cut-offs, two timepoints may comprise men at the same point in their supervision. For example, both Time 1 and Time 2 consist of men that are about 3 months into their community supervision. However, the purpose of the study is to examine changes in typologies based on scores across assessment occasions, as opposed to examining change in typologies as a direct function of time on supervision. Research examining the optimal timing for typological development and follow-up timepoints is warranted.

There were also some variables that were not available in the administrative dataset. For example, the current study was unable to include sentence type as an auxiliary variable to examine potential typological differences between those on stand-alone community supervision in comparison to those on supervision post-release from a provincial correctional facility. As such, future research is encouraged to examine whether there are differences in typological membership and profile transitions between these two groups. The current study was also unable to look at item-level SPIn data, and instead utilized SPIn domain level information, comprised of several items, to inform treatment typologies. The influence of any specific item was masked by the combined domain total score. Future research should examine SPIn item-level data to see if there are specific items that cluster together that are most predictive of reoffending outcomes.

A final limitation is the lack of information available on programming and frequency of contact with a supervising officer. Programming experienced in the community can influence profile membership changes (e.g., programming offered to higher risk men may assist with reducing various criminogenic needs and increasing various strengths over time). Although this data was not available, it was determined that in Alberta, programming is often completed by non-government organizations (Programs and Policy Development unit from Alberta Justice and Solicitor General, personal communication, January 28, 2020). As such, there may be differences among jurisdictions due to resources and client needs. Future research should examine whether there are jurisdictional differences in typological membership and changes in typological membership over time, as well as whether frequency of contact with a supervising officer influences changes in typological membership over time.

### Conclusion

This study was the first to incorporate a combination of risk and strength factors to inform treatment typologies among men on community supervision. Results indicated that those who were low risk were split into two profiles—one with high strengths and one with low strengths. Higher risk cases were split into an aggressive and a non-aggressive profile, each with complex needs and low strengths, and these profiles were most likely to reoffend. The findings highlight the importance of theoretical integration—that is, combining risk- and strength-based perspectives to understand the variability among men on community supervision, and can help inform effective service delivery, including programming and supervision practices.

## Notes

1 In Canada, people who are sentenced for 2 years less a day, or less, enter the provincial correctional system, whereas those who are sentenced to 2 years or more are involved in the federal correctional system.

2 Given that those who reoffended prior to receiving three assessments were excluded (i.e., a technical violation or new charge resulting in recontact with correctional services in the province of Alberta), a supplemental LPA was conducted on all men who had a complete SPIn Full Assessment within 90 days of start of supervision. This was conducted to see how the typological structure changes when including men with less than three assessments and who recidivate prior to 14 months. Overall, results indicated that five similar profiles emerged (for more details, see Wanamaker (2020)).
